# Two Monoclonal Antibodies That Specifically Recognize *Aspergillus* Cell Wall Antigens and Can Detect Circulating Antigens in Infected Mice

**DOI:** 10.3390/ijms23010252

**Published:** 2021-12-27

**Authors:** Xihua Lian, Stephen Chambers, John G. Lewis, Amy Scott-Thomas, Madhav Bhatia

**Affiliations:** 1 Department of Pathology and Biomedical Science, University of Otago, Christchurch 8011, New Zealand; xihua.lian@postgrad.otago.ac.nz (X.L.); steve.chambers@otago.ac.nz (S.C.); john.lewis@otago.ac.nz (J.G.L.); amy.scott-thomas@otago.ac.nz (A.S.-T.); 2 Canterbury Health Laboratories, Christchurch 8011, New Zealand

**Keywords:** monoclonal antibody, *Aspergillus* antigens, invasive aspergillosis, detection assay

## Abstract

Invasive aspergillosis (IA) is a life-threatening disease mainly caused by *Aspergillus fumigatus* and *Aspergillus flavus*. Early diagnosis of this condition is crucial for patient treatment and survival. As current diagnostic techniques for IA lack sufficient accuracy, we have raised two monoclonal antibodies (1D2 and 4E4) against *A. fumigatus* cell wall fragments that may provide a platform for a new diagnostic approach. The immunoreactivity of these antibodies was tested by immunofluorescence and ELISA against various *Aspergillus* and *Candida* species in vitro and by immunohistochemistry in *A. fumigatus* infected mouse tissues. Both monoclonal antibodies (mAbs) showed intensive fluorescence with the hyphae wall of *A. fumigatus* and *A. flavus*, but there was no staining with other *Aspergillus* species or *Candida* species. Both mAbs also showed strong immunoreactivity to the cell wall of *A. fumigatus* hyphae in the infected liver, spleen and kidney of mice with IA. The antigens identified by 1D2 and 4E4 might be glycoproteins and the epitopes are most likely a protein or peptide rather than a carbohydrate. An antibody-based antigen capture ELISA detected the extracellular antigens released by *A. fumigatus*, *A. flavus*, *A. niger* and *A. terreus*, but not in *Candida* species. The antigen could be detected in the plasma of mice after 48 h of infection by double-sandwich ELISA. In conclusion, both 1D2 and 4E4 mAbs are potentially promising diagnostic tools to investigate invasive aspergillosis.

## 1. Introduction

Invasive aspergillosis (IA) is an opportunistic infection that can be acute, rapidly progressive, and life-threating in an immunocompromised host. It occurs worldwide and, overall, more than 90% of cases are caused by *Aspergillus fumigatus* [1,2,3], *Aspergillus flavus* is common in some geographic regions, particularly in Asia [4]. Infection is usually through airborne conidia that may infect the sinuses, lungs or both structures. After germination, *Aspergillus* species form hyphae that spread locally, but can also cross tissue planes, invade blood vessels and metastasize through the blood stream to other organs such as the brain and skin. Dissemination may be inhibited by local platelet activation and thrombosis, although this is compromised in patients with thrombocytopenia [5,6]. The clinical diagnosis of IA is extremely difficult because IA lacks specific clinical features. Symptoms such as cough, fever and dyspnea occur in IA but have many other causes in the immune suppressed host [7]. Fever is a common clinical feature of IA in the immune suppressed patient; however, those patients are not responsive to antibiotic therapy directed against bacterial pathogens. Despite the prophylaxis and treatment of IA, the outcome of cases is poor, and the mortality rate is reported to be up to 90% if the diagnosis is delayed [8,9,10,11,12].

The current methods for the diagnosis of IA lack sufficient specificity and sensitivity to make early and accurate diagnosis reliable. The current gold standard for IA diagnosis is observation of *Aspergillus* in biopsy tissue samples, or a positive culture of *Aspergillus* from a specimen taken from a normally sterile site [13,14]. The hazard of the invasive procedures needed to get tissue specimens severely limits the usefulness of these methods in immunosuppressed patients [15,16]. Serological testing is of very limited value in acute infection because of the time taken for an antibody response and this is unreliable in immunocompromised patients [15]. Conventional imaging examinations such as CT and MRI, have high resolution but are unable to reliably distinguish lesions caused by fungal infections from other types of focal lesion [17]. The polymerase chain reaction (PCR) test is not universally employed in IA diagnosis owing to a lack of standardization although it has been included as a criterion for probable invasive pulmonary aspergillosis in the most recent European Organization for Research and Treatment of Cancer and the Mycoses Study Group Education and Research Consortium (EORTC/MSGERC) guidelines [13]. Less invasive tests such as galactomannan (GM) and (1-3)-β-d-glucan detection, may provide evidence of *Aspergillus* infection [13,16,17,18,19,20]. Of these, the commercial immunoenzymatic double-sandwich microplate assay, called Platelia *Aspergillus* assay (Bio-Rad, Marnes-La-Coquette, France), has become widely used for the detection of *Aspergillus* GM antigen in serum and bronchoalveolar lavage fluid. However, this assay requires serial testing in serum, and lacks sensitivity and may give false-positive results in patients treated with piperacillin-tazobactam [21,22,23].

Because of these deficiencies in the current tests, we have developed two new monoclonal antibodies (mAbs) that may provide a platform for new tests for IA. In this study, we report the characterization of two new mAbs against *A. fumigatus* cell wall antigens and their usefulness as potential diagnostic tools for IA.

## 2. Results

### 2.1. mAbs Reactivity and Specificity

#### 2.1.1. ELISA

**Figure 1:**
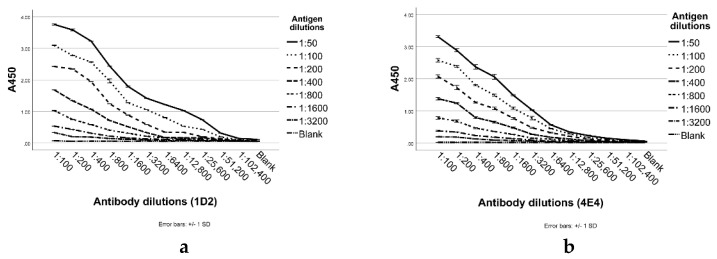
Purified 1D2 and 4E4 detect immobilized *Aspergillus* *fumigatus* cell wall fragments by ELISA. A range of concentrations (0.0275–1.76 µg/mL) of *A.* *fumigatus* cell wall antigens were coated on the microtiter plate. After blocking and washing, the immobilized fragments were detected by adding purified 1D2 (**a**) or 4E4 (**b**) with serial dilutions (0.0098–10 µg/mL) and goat anti-mouse IgM-HRP (1:2000). A450: Absorbance at 450 nm.

The production of mAbs in mice immunized with *A. fumigatus* cell wall soluble fragments resulted in five antibody positive wells but only two displayed competitions with soluble fragments. These two hybridoma clones designated 1D2 and 4E4 were both isotyped as IgM kappa. Dilutions of both antibodies recognized cell wall fragments (CWFs) of *A. fumigatus* by ELISA (Figure 1) even at low coating concentrations of antigen.

#### 2.1.2. Immunofluorescence and Immunohistochemistry

**Figure 2:**
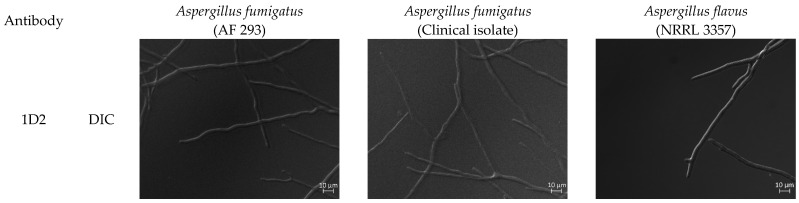

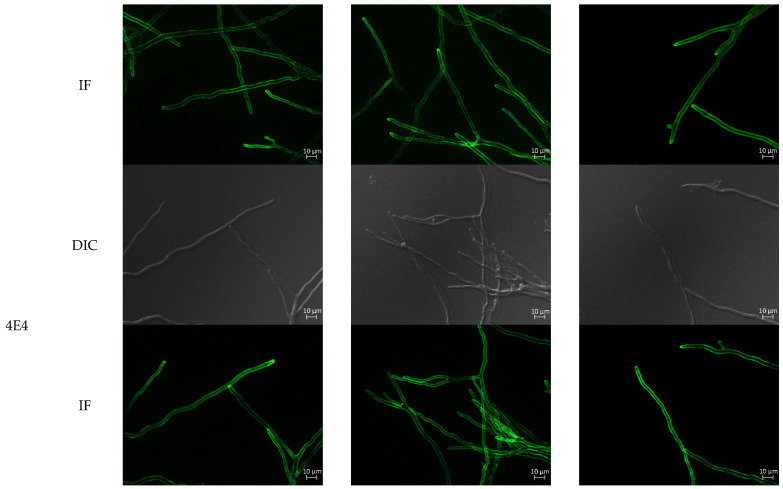
mAbs 1D2 and 4E4 stain *Aspergillus fumigatus and Aspergillus flavus.* 1D2 and 4E4 identified the cell wall antigens of *A. fumigatus* including ATCC strain and clinical strains, and *A.* *flavus*. All of them showed intense fluorescence, particularly on the hyphal tips. DIC: differential interference contrast | IF: immunofluorescence.

Hybridoma supernatants 1D2 and 4E4 showed intense fluorescence on the hyphal wall of *A. fumigatus* (AF 293 strain as well as clinical isolates) and *A. flavus*, suggesting that both antibodies recognize the surface antigens, especially on the hyphal tips (Table 1, Figure 2). No fluorescence was detected using the control mouse IgM isotype antibody 6B10. In addition, both 1D2 and 4E4 showed detectable fluorescence on the cell wall of early geminated conidia but lacked staining on the resting conidia and did not recognize any other fungi tested (Table 1, Supplementary Figures S1 and S2).

**Figure 3:**
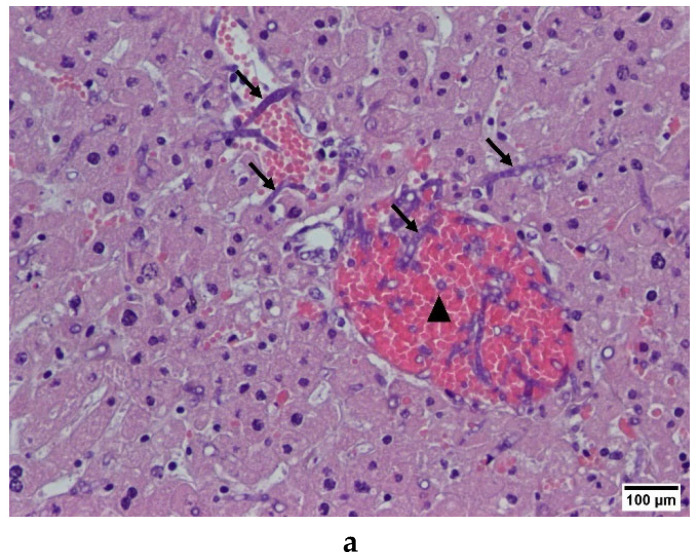

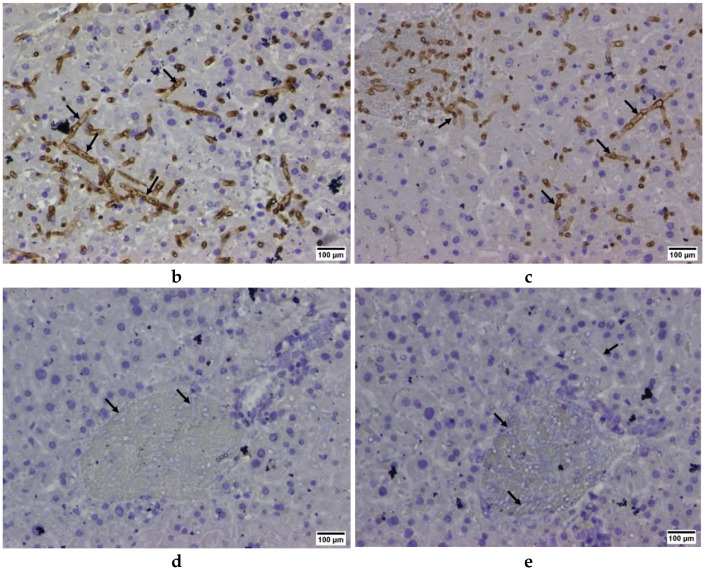
*Aspergillus* mAbs 1D2 and 4E4 showed immunohistochemical staining in formalin-fixed paraffin liver sections from *Aspergillus fumigatus* infected mice. (**a**) Haematoxylin and eosin staining showed that the hepatic lobule structure was disordered with radial structure appearing, the central hepatic vein was expanded, congested and full of red blood cells (triangles); the hepatocytes were swollen and partly fused with a shrinking or disappearing nucleus; the *A. fumigatus* hyphae infiltrated the blood vessels (arrow), with no obvious neutrophil infiltration. (**b**–**e**) Immunohistochemistry staining indicated both mAbs 1D2 (**b**) and 4E4 (**c**) showed significant signals on the hyphal wall (black arrow) of *A. fumigatus*. There was no immunoreactivity observed in isotype group (**d**) and blank control (**e**) group.

We also found, by immunohistochemistry, both 1D2 and 4E4 recognize the cell wall of *A. fumigatus* hyphae in infected liver, spleen, and kidney from IA mice (Figure 3 and Figure S3), indicative that both mAbs can recognize the cell wall antigens of *A. fumigatus* after they germinate in mouse tissue.

### 2.2. Characterization of Antigens Recognized by mAbs 1D2 and 4E4

**Figure 4:**
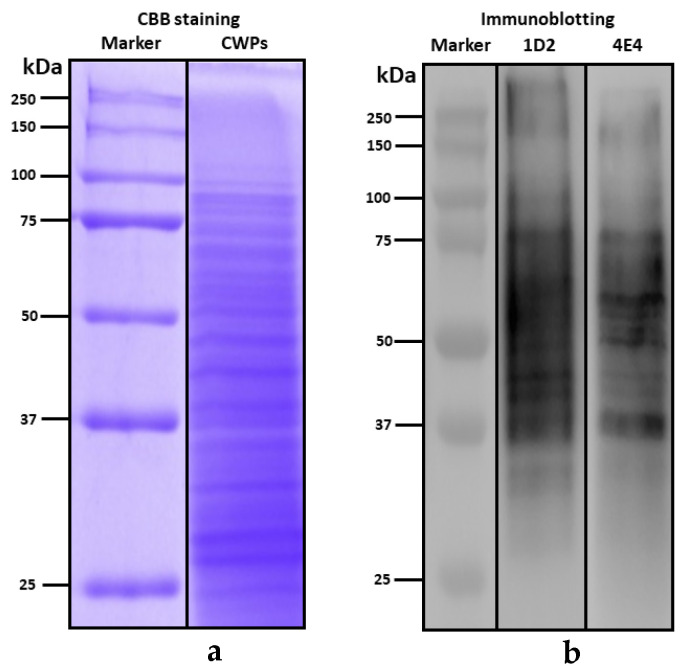
Characterization of *Aspergillus fumigatus* antigens recognized by mAbs 1D2 and 4E4. The *A. fumigatus* cell wall proteins (CWPs) were separated by SDS-PAGE and stained by Coomassie Brilliant Blue (CBB) (**a**). By Western blotting (**b**), both mAb 1D2 and 4E4 showed a diffuse signal to *A. fumigatus* CWPs with molecular weight exceeding 37 kDa, particularly some visible bands with molecular weight between 37 kDa and 75 kDa.

**Figure 5:**
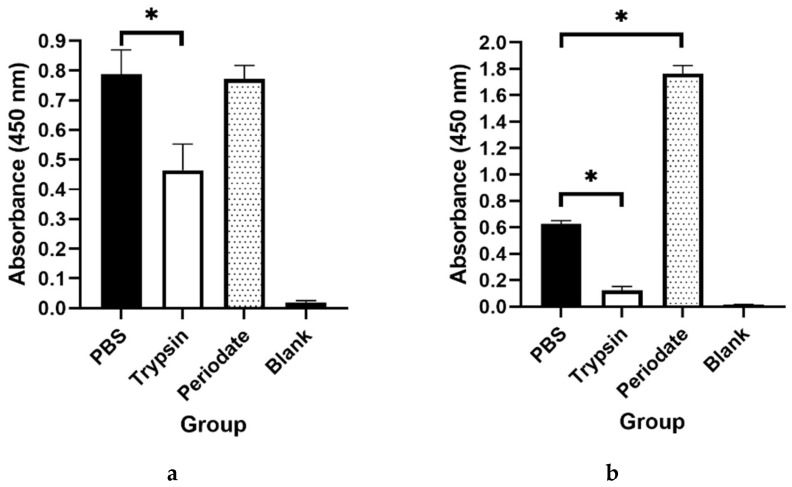
Investigation of trypsin or periodate-treated *Aspergillus fumigatus* antigens. For mAb 1D2 (**a**), the absorbance at 450 nm of the trypsin-treated group was significantly lower than the untreated (PBS) group, but there is no difference between the periodate-treated group and the untreated group. For mAb 4E4 (**b**), trypsin treatment reduced the absorbance compared to the untreated group, but periodate treatment significantly upregulated the epitopes binding. An asterisk denotes a statistically significant difference between the treated group and the untreated group (*p* < 0.05).

Both Western blotting (Figure 4) and indirect ELISA (Figure 5) showed positive binding of mAbs (1D2 and 4E4) to *A. fumigatus* cell wall-related proteins (CWPs), indicating that the antigens were most likely a glycoprotein. After separation by SDS-PAGE (Figure 4a), both 1D2 and 4E4 supernatant displayed a diffuse mode to *A. fumigatus* CWPs with molecular weight more than 37 kDa, especially those visible protein bands between 37 kDa and 75 KDa (Figure 4b). Moreover, the epitope recognized by antibody 1D2 was sensitive to trypsin (*p* < 0.05) but not to periodate (*p* > 0.05) (Figure 5a). Similarly, the epitope identified by antibody 4E4 was decreased by trypsin treatment (*p* < 0.05). However, the absorbance increased significantly (*p* < 0.05) in the periodate group compared to the untreated (PBS) group (Figure 5b).

Furthermore, the binding of either 1D2 or 4E4 to the immobilized *A. fumigatus* cell wall fragments (CWFs) was not inhibited by the addition of galactomannan (GM) up to the high concentration of 200 μg/mL (*p* > 0.05, Supplementary Figure S4), which further confirms that both 1D2 and 4E4 do not identify the carbohydrate epitopes located on the GM. 

The inhibition percentages of biotin-conjugated 1D2 and biotin-conjugated 4E4 with unlabelled 1D2 and 4E4 are shown in Table 2. It shows that the binding of both 1D2-biotin and 4E4-biotin to the immobilized antigens are relatively inhibited (inhibition percentage was between 25% and 75%) by homologous and heterologous antibodies, suggesting that they may have similar binding to the cell wall and recognize similar or spatially overlapping antigen epitopes leading to interactive competition.

### 2.3. Development of Antibody-Based Antigen Capture Double-Sandwich ELISA

#### 2.3.1. Optimization of the Double-Sandwich ELISA

**Figure 6:**
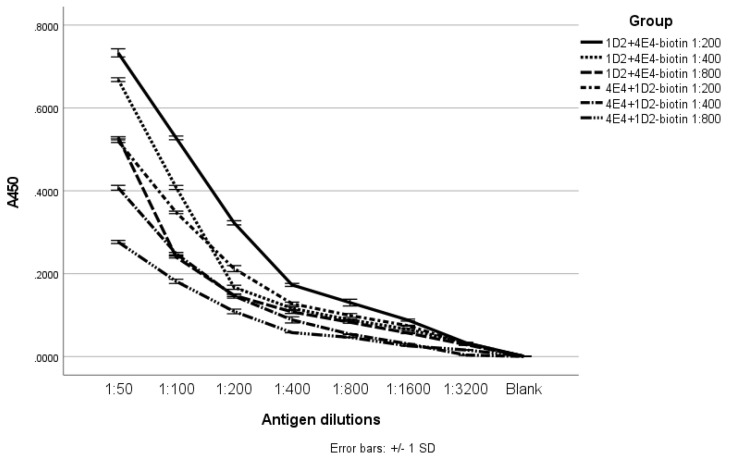
Sandwich ELISA optimization. The combination of 1D2 (5 µg/mL) as the capture antibody and 4E4-biotin (5 µg/mL) as the detection antibody showed the best absorbance with the various cell wall antigen concentrations (solid line).

We found for the sandwich ELISA that the combination of 1D2 as the capture antibody and 4E4-biotin diluted at 1:200 (5 µg/mL) as the detection antibody showed optimal absorbance with the various cell wall antigen concentrations (Figure 6).

#### 2.3.2. The Application of Monoclonal Antibody-Based Antigen-Capture Double-Sandwich ELISA

**Figure 7:**
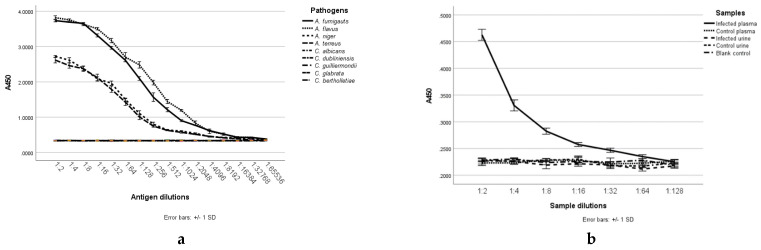
The double-sandwich ELISA detects only *Aspergillus* fungal antigens in culture filtrate dilutions (**a**). It also detects circulating antigens in dilutions of plasma but not urine in mice with invasive aspergillosis (**b**).

The optimized sandwich ELISA assay was used to test fungal culture filtrates. Both *A. fumigatus* and *A. flavus* displayed strong positive responses with the antibodies at dilutions of greater than 1:4096. Dilutions of culture filtrates of *A. niger* and *A. terreus* also showed positive signals, but other fungal culture filtrates did not react in the sandwich ELISA (Figure 7a).

ELISA testing of plasma samples from the *A. fumigatus*-infected mice found a positive signal in dilutions of up to 1:32 (Figure 7b). There was no apparent difference between urine from the infected and non-infected control group of mice.

## 3. Discussion

Invasive aspergillosis (IA) is one of the most severe invasive fungal infection diseases and has a high rate of morbidity and mortality particularly in immunosuppressed patients [1,2]. The number of IA patients is gradually increasing due to an increase in the use of immunosuppressive agents and the immune suppressive effects of severe infections such as COVID [24,25,26,27]. The early and accurate diagnosis of IA is of critical importance and can guide the early anti-fungal therapy, which is beneficial for IA survival [8,9,10,11,12].

In this study, we report our results with two novel monoclonal antibodies (mAbs) against *A. fumigatus* cell wall antigens from two hybridoma clones. The sensitivity and reactivity of the mAbs were determined by ELISA, immunofluorescence, and immunohistochemistry. The immunofluorescence studies showed that the antibodies were highly sensitive and specific to *A. fumigatus* and *A. flavus* hyphae but did not cross-react with other *Aspergillus* species and *Candida* species. The immunohistochemistry studies showed that these two mAbs can be used as probes to detect *A. fumigatus* hyphae in infected tissues in mice. The antigens identified by 1D2 and 4E4 were tested to be glycoprotein by Western blotting and ELISA, and the epitopes are most likely a protein or peptide rather than a carbohydrate. We paired 1D2 and 4E4-biotin in double-sandwich ELISA, which was able to detect the antigen in extracellular fractions in culture media of *A. fumigatus*, *A. flavus*, *A. niger* and *A. terreus*. Furthermore, this pair could detect the antigenemia in mice two days after inoculation with *A. fumigatus* conidia.

Both 1D2 and 4E4 identified the surface antigens presented on *A. fumigatus* hyphae by immunofluorescence, as well as *A. flavus* hyphae, but not other *Aspergillus* species or *Candida* species. Immunohistochemistry studies demonstrated that both mAbs also reacted with hyphal wall antigens expressed during infection of multiple tissues of immunosuppressed mice indicating that the antigens were expressed in differing nutritional environments [28,29]. The antigens detected by these antibodies were not detectable in *A. fumigatus* resting conidia but were seen in very short hyphae that were still attached to the conidia, suggesting that these antigens were expressed very early in the growth cycle. The antigens also appeared to be incorporated along the length of the hyphal cell wall and were seen most intensely in the growing tip of the hyphae, suggesting that they were continually produced during the growth phase of hyphae.

*Aspergillus* cell wall comprises mainly of polysaccharides, such as chitin, glucans, and galactomannan, as well as glycoproteins [30,31]. The positive diffuse reaction pattern of *A. fumigatus* cell wall-related proteins (CWPs) and mAbs in immunoblotting and positive signals of ELISA, as well as the non-competition between the GM and immobilized CWPs, all indicate that the antigens recognized by 1D2 and 4E4 are more likely to be glycoproteins rather than a GM-related polysaccharide. This is supported by our findings that the antigens recognized by both 1D2 and 4E4 were sensitive with protease, indicating that the epitopes are likely to be a protein or peptide. Conversely, the periodate-treated samples had similar absorbance to the untreated group, indicating that 1D2 epitope is not likely a carbohydrate [29,32]. Furthermore, the multiple protein bands with molecular weight between 37 kDa and 75 kDa also demonstrate that the epitopes recognized by 1D2 and 4E4 exist in many different glycoproteins. Both assays were performed and treated similarly with other studies [29,32,33] but showed different results; therefore, we believe that these two novel mAbs are distinctive with those that have been previously reported [29,32,33].

There was some evidence that the two mAbs recognized different antigens. Firstly, the diffuse immunoblotting signals with some visible bands between 1D2 and 4E4 were different, albeit the similar molecular weight. Secondly, the absorbance of periodate-treated 4E4, but not 1D2, was significantly higher than the untreated group. A possible explanation for this observation is that some of the 4E4 epitopes are masked by the glycans modification on the glycoprotein and periodate oxidation alters the tertiary structure of the glycoprotein, exposing additional epitopes for 4E4 binding [29,32]. In addition, the competitive ELISA using the two mAbs demonstrated that there was some competition between 1D2 and 4E4, suggesting that these two antibodies recognize similar or spatially overlapping epitopes of the respective antigens.

As the 1D2 antibody gave higher absorbance on the indirect assay, this antibody was then used as a capture antibody and 4E4-biotin as the detection antibody in a double-sandwich ELISA test for the *Aspergillus* antigen. This antibody pair was able to detect the antigen in extracellular fractions in culture media from *A. niger* and *A. terreus* as well as *A. fumigatus*, *A. flavus*, although the amount of the antigen in the supernatant of *A. niger* and *A. terreus* was lower than *A. fumigatus* and *A. flavus*. These results demonstrate that significant amounts of the antigen are produced and shed by all four *Aspergillus* species into the culture media, although it was not detectable in hyphae of cultured *A. niger* and *A. terreus* by immunofluorescence. Possible reasons include differing concentrations of the antigens in the hyphal wall, masking of the epitopes within the cell wall or different configurations or orientation of the epitopes in the hyphal wall following shedding into the medium [29,34].

Analysis of blood and urine samples harvested on day two from the immunosuppressed mouse model of IA demonstrated that the antigen shed from hyphae reached the blood stream but was not detected in urine. These findings are similar to those reported by others in both human and animal studies of IA, although GM has been reported to be present in urine [35,36]. These indicate that the mAb-based approach has practical usefulness in monitoring IA progression or treatment effect or screening those individuals with a high-risk of *Aspergillus* infection.

In summary, the mAbs 1D2 and 4E4 described in this study are capable of detecting *Aspergillus* antigens and have important characteristics that show promise for clinical applications. Both antibodies were able to react with *A. fumigatus* antigens expressed during the invasion of mouse tissues and may have diagnostic potential when coupled with immunofluorescence, immunohistochemical or other techniques. The double-sandwich ELISA using 1D2 and 4E4-biotin that successfully detected antigen in blood has promise as the foundation for a diagnostic assay for IA. Future studies will explore the value of these antibodies in the diagnosis of IA in human disease.

## 4. Materials and Methods

### 4.1. Microorganism Strains and Culture Conditions

The *A. fumigatus* (AF 293), *A. flavus* (NRRL 3357) used in this study were obtained from the University of Wisconsin–Madison, USA. Another two *A. fumigatus* clinical strains, *Aspergillus* *terreus*, *Aspergillus* *niger*, *Cunninghamella bertholletiae, Rhizopus microsporus, Candida albicans*, *Candida dubliniensis*, *Candida guilliermondii*, *Candida glabrata, Candida parapsilosis, Candida tropicolis* and *Candida lusitaniae* strains were obtained from Canterbury Health Laboratories, Christchurch, New Zealand. These microorganisms originated from clinical isolates recovered from clinical specimens. All fungi were cultured on Sabouraud dextrose agar (SDA) plates or in Sabouraud dextrose (SD) liquid media at 37 °C, unless otherwise specified.

### 4.2. Preparation of A. fumigatus Conidia Suspension

*A. fumigatus* (AF 293) was recovered from −80 °C glycerol stock and streaked on SDA plates. After culturing 3 to 5 days, the conidia from a single colony were streaked on a new SDA plate and grown for another 3 to 5 days until a sufficient amount of conidia were observed. The conidia were harvested and collected by pouring through 8 layers of sterile Miracloth (Merck, Darmstadt, Germany). The conidia suspension was centrifuged at 5000× *g* three times, 5 min for each. The supernatant was discarded, and the conidia pellet was resuspended with phosphate-buffered saline (PBS). The concentration of the suspension was adjusted to 1 × 10^6^ conidia/mL and 2 × 10^6^ conidia/mL using a haemocytometer and stored at 4 °C.

### 4.3. Preparation of Aspergillus Cell Surface Antigens and Extracellular Antigens

For the preparation of cell surface antigens, the *A. fumigatus* (AF293) spores from a single colony were inoculated to SD liquid media and cultivated for 5 to 7 days in a shaking incubator until mycelia were observed. The mycelia were harvested by filtration through sterile Miracloth and placed into a sterile tube and washed thoroughly with PBS, followed by resuspension in PBS and disrupted by sonication at 4 °C. The broken hyphae mixture was centrifuged for 15 min (10,000× *g*, 4 °C), the supernatant containing the soluble *Aspergillus* cell wall fragments (CWFs) was collected and stored at −80 °C until they were used. 

Cell wall-related proteins (CWPs) of *A. fumigatus* were precipitated from the *A. fumigatus* CWFs by addition of 9 volumes of cold ethanol overnight at −20 °C. After centrifugation for 10 min (13,000× *g*, 4 °C), the precipitated proteins were rewashed three time with cold 90% ethanol and resuspended with a small volume of Tris-HCl buffer (pH 7.4) and stored at −80 °C.

The extracellular antigens were collected as described previously with minor revisions [29,37]. In brief, 1 mL of 2 × 10^6^ conidia/mL of *A. fumigatus*, *A. flavus*, *A. niger*, and *A. terreus* and other *Candida* species were cultured in 200 mL of SD media on a shaking incubator for two weeks. The extracellular antigens were collected by precipitation with cold ethanol as mentioned above. The extracellular antigens were finally resuspended with Tris-HCl buffer (pH 7.4) and stored at −80 °C. Protein concentrations were tested by Bradford assay, as previously described [38]. The concentrations of various culture filtrates were adjusted to the same for comparison. 

### 4.4. The Production and Screening of mAbs (1D2 and 4E4) against A. fumigatus Cell Wall Antigen Extracts

Three female Balb/c mice were immunized intraperitoneally with 50 µg *A. fumigatus* soluble cell wall fragments (CWFs) in 200 µL of PBS emulsified with an equal volume of complete Freund’s adjuvant at 4-week intervals and using 100 µL emulsion per mouse. One week after the 4th injection, blood from tail vein tipping was tested for antibody production using *A. fumigatus* CWFs-coated plates. Splenic lymphocytes from the best responder were then fused with FOX-NY myeloma cells (CRL-1732) at a ratio of 5:1 using 50% polyethylene glycol and plated out in 96-well culture plates at 100,000 cells/well, as previously described [39]. After 12 days culture at 37 °C and 5% CO_2_, supernatants from the hybridoma cells were screened using *A. fumigatus* CWFs-coated microtiter plates. Briefly, culture supernatants were transferred to antigen-coated and blocked plates and following incubation and washing. The bound antibody was detected with anti-mouse Ig-peroxidase. Antibody positive wells were then retested in the presence and absence of excess soluble *A. fumigatus* antigens to ascertain competition and by inference specificity. Cells in these corresponding positive specific wells were cloned to single clones by limiting dilution in 96-well plates using Balb/c spleen cells as a feeder layer (100 × 10^3^ cells/well). Supernatants from the cloning were retested and a positive single clone selected and this hybridoma was expanded. 

### 4.5. Monoclonal Antibodies Purification and Biotinylation

Both mAbs 1D2 and 4E4 were purified from the hybridoma supernatant using a mouse IgM purification resin column (LT-145KIT, LigaTrap) following the instructions, as specified by the manufacturers. The concentration of recovered antibody was determined by testing the absorbance of UV visible spectrophotometer (Agilent Technologies, Santa Clara, CA, USA) at 280 nm. The concentration was adjusted to 1 mg/mL and stored at −20 °C. The purity of antibody was confirmed by implementing sodium dodecyl sulphate polyacrylamide gel electrophoresis (SDS/PAGE) and gel dyeing by Coomassie Brilliant Blue (CBB) R-250 dye (BDH Laboratories, London, UK) (Supplementary Figure S5).

After purification, both mAbs were biotinylated using commercial EZ-Link Sulfo-NHS-Biotin (Thermo Fisher Scientific, Waltham, MA, USA). Briefly, both 1D2 and 4E4 were dialysed to remove any buffer-containing primary amines and adjusted to 1 mg/mL with PBS. 50-fold molar excess of EZ-Link Sulfo-NHS-Biotin reagent was added and incubated for 30 min at room temperature (RT) in a reaction vial with continual stirring. The labelled antibody was dialysed exhaustively again to remove the nonreacted biotin against PBS. The concentration of biotinylated antibody was adjusted to 1 mg/mL. The biotinylating was confirmed by direct ELISA via reacting with the immobilized *A. fumigatus* antigen coated on the microtiter plate and detected by streptavidin-HRP (Jackson Immuno Research, Philadelphia, PA, USA).

### 4.6. Enzyme-Linked Immunosorbent Assays (ELISAs)

#### 4.6.1. Monoclonal Antibodies Reactivities to *A. fumigatus* Antigens

For the indirect ELISA, the process was similar to that described by Schubert et al. [29]. Briefly, various concentrations of *A. fumigatus* CWFs in PBS were coated overnight on microtiter plate wells (Corning, New York, NY, USA). The following day, the plate was washed with PBS containing 0.1% Tween 20 (PBST) and the wells were blocked with 3% BSA in PBST. Then, serial dilutions of purified 1D2 and 4E4 (from 1:100 to 1:102,400) were added to each well (100 μL) for 1 h with the control being an unrelated IgM isotype antibody (6B10) [40]. After washing, 100 μL of goat anti-mouse IgM-HRP (Abcam, Cambridge, UK) diluted at 1:2000 was added to each well, followed by washing and detection with tetramethybenzidine (TMB) substrate. The reaction was stopped by the addition of 100 μL of 1 M hydrochloric acid/well and the absorbance was read by a microplate spectrophotometer at 450 nm (Thermo Fisher Scientific, Waltham, MA, USA). With the exception of coating and washing, all other dilution steps in the ELISAs were in PBST containing 3% BSA. All laboratory ELASAs in this study were performed at RT with triplicate determinations unless otherwise specified. The optimal reaction concentrations of all antibodies were determined by titration.

#### 4.6.2. Characterization of Antigen Epitopes Recognized by Monoclonal Antibodies 

To analyse the characterizations of antigens recognized by these two mAbs and whether the epitopes were a carbohydrate or a protein (peptide), we treated the *A. fumigatus* cell wall-related proteins (CWPs) with periodate and trypsin to oxidize the glycans or digest the proteins. Specifically, *A. fumigatus* CWPs were coated on a flat-bottom plate overnight. The coated samples were treated with 100 μL/well of sodium *meta*-periodate (20 mM NaIO_4_ in 50 mM sodium acetate buffer, pH 4.5) at 4 °C or trypsin (1 mg/mL) at 37 °C, respectively, overnight, as tested previously with some changes [32]. After washing and blocking as described above, 100 μL/well of purified 1D2 and 4E4 (2.5 μg/mL) were added and followed by detecting, as mentioned above. Untreated CWPs were set as the positive control and PBS as the blank control.

The competitive ELISA was performed using *A. fumigatus* CWFs coated on to microtiter plate wells as described above. After washing and blocking, 50 μL/well of galactomannan (1 mg/mL) with various dilutions (from 1:5 to 1:800) were added, immediately followed by addition of 50 μL/well of purified 1D2 or 4E4 (5 μg/mL) respectively and incubated 1 h. After further washing, the signals were detected as described above.

Another competitive ELISA using immobilized CWFs was used to differentiate epitopes identified by 1D2 and 4E4, in a similar manner to that described by others [41]. Briefly, *A. fumigatus* CWFs were coated overnight on to microtiter plate wells (0.022 µg/well). After washing and blocking, 100 μL/well of purified and serially diluted mAbs 1D2 and 4E4 were added and incubated overnight at 4 °C. The following day, 100 μL of biotinylated antibody, either 1D2 or 4E4 (2.5 µg/mL) was added for 1 h to each well. The signals were detected by addition of 100 μL/well of streptavidin-HRP diluted at 1:1000 for a further hour and followed by substrate addition and processing as described above. These crossover experiments with combinations of capture and biotinylated antibodies allowed calculation of the relative inhibition percentage based on comparison of A450 nm measurements. According to Hao et al. [41], antibodies are strongly competitive if the inhibition percentage was greater than 75% and between 25% and 75% deemed as relatively competitive, and less than 25% as non-competitive.

#### 4.6.3. Development of Antibody-Based Antigen Capture Double-Sandwich ELISA

We also established and optimized a double-sandwich ELISA similar to that described by others [41] in order to detect extracellular antigens secreted by cultures of different pathogens adjusted to similar protein concentrations. Briefly, 100 μ of purified capture antibody either 1D2 or 4E4 (5 µg/mL in PBS) was coated on a flat-bottomed microtiter plate at 4 °C. After washing and blocking, 100 μL of samples containing the antigens were serially diluted and added to each well and incubated for 1 h and following washing. Next, 100 μL/well of serial dilutions of biotin-labelled 1D2 or E4E were added for a further 1 h, after which the bound biotinylated antibody was detected with streptavidin-HRP with further processing as described above. The optimized double-sandwich ELISA system was then used to test whether or not it could detect released antigens in dilutions of plasma from *A. fumigatus*-infected mice compared to non-infected controls.

### 4.7. Western Blotting

Western blotting technique was used to verify whether these two monoclonal antibodies recognize the protein antigens or not. *Aspergillus* cell wall-related proteins (CWPs) samples with loading buffer were denatured at 95 °C for 10 min. The CWPs samples were separated in 10% SDS-PAGE and the immigration was completed in electrophoresis buffer (Tris-glycine-SDS). The CWPs were visualized by Coomassie Brilliant Blue (CBB) staining or transferred to a PVDF membrane in transfer buffer (Tris-glycine-methanol). The membrane was washed with Tris buffered saline (TBS) with 0.1% tween 20 and blocked with 5% trim milk for 1 h on the rocker. Afterward, the membrane was incubated with 1D2 or 4E4 supernatant (1:200) in 1% BSA at 4 °C overnight on the rocker. With 5 times (10 min each) washing, anti-mouse IgM-HRP secondary antibody (Abcam, Cambridge, UK) diluted at 1:10,000 was applied and incubated for 1 h on a rocker. After washing 5 times, the membrane was developed by incubation with chemiluminescent substrate (PerkinElmer, Waltham, MA, USA) and imaged by Alliance Q9 Advanced (Uvitec, Cambridge, UK).

### 4.8. Immunofluorescence

Immunofluorescence microscopy experiments were performed as previously described with some minor changes [42]. In short, 10 μL/well of conidia suspension (1 × 10^6^ conidia/mL) were seeded in Lab-Tek 8-well chamber slide (Thermo Fisher Scientific, Waltham, MA, USA) with 350 µL of SD media. After incubation at 37 °C for 16 h to germinate, the media was aspirated, and the slide was air-dried. The samples were fixed by adding 200 µL/well of 4% formaldehyde for 20 min and rinsed three times with PBS. After blocking with 3% BSA in PBS for 1 h, 200 µL/well of hybridoma cells supernatant containing mAbs (diluted at 1:10) was applied and incubated for 1 h. Followed by incubating samples with 200 µL/well of goat anti-mouse IgM secondary antibody-FITC (Invitrogen, Waltham, MA, USA) diluted at 1:200 for 1 h in the dark. Control groups were incubated with blocking buffer or isotype IgM (6B10) but were otherwise treated the same [40]. The samples were mounted and observed with an epifluorescence microscope (Carl Zeiss, Oberkochen, Germany).

### 4.9. Immunohistochemical Staining

For immunohistochemistry, infected tissues from IA mice were fixed in 10% formalin overnight and embedded in paraffin, cut to 4 µm, affixed onto an adhesive slide (Trajan Scientific and Medical, Ringwood, Australia) and dried for about 20 min. After deparaffinization and rehydration in xylene and serial dilutions of ethanol, the antigen retrieval was completed in the pressure cooker (Hawkins, Mumbai, India) with 10 mM sodium citrate buffer (pH 6.0). The tissue samples were then permeabilizated by PBS with gelatin (0.2% *w*/*v*) and Triton (0.25% *v*/*v*). Afterward, the samples were blocked in 5% BSA in permeabilization solution at RT for 1 h and incubated with antibody supernatant (diluted at 1:10) overnight at 4 °C. The next day, after three 10-min washes in PBS, the endogenous peroxidase of the tissue was inactivated by 3% hydrogen peroxide before incubation with anti-mouse IgM-HRP (Abcam, Cambridge, UK) for 1 h at RT. The samples were developed with 3,3’-diaminobenzidine (DAB) solution (Agilent Technologies, Santa Clara, CA, USA) for 5 min and counterstained with haematoxylin for 1 min at RT. Finally, the samples were dehydrated and permanently mounted and observed under the light microscope (Olympus, Tokyo, Japan). Control groups were incubated with blocking buffer or isotype IgM (6B10) but were otherwise treated the same.

### 4.10. Establishment of Invasive Aspergillosis Mouse Model

All animal work was approved by the Animal Ethics Committee of the University of Otago (AUP-20-88) in compliance with the New Zealand animal welfare regulation. Male Balb/c mice aged 10–12 weeks were used to establish the invasive aspergillosis animal model. Immunocompromised mouse model was induced by intraperitoneal injection of 150 mg/kg of cyclophosphamide (Baxter Healthcare Limited, Auckland, New Zealand) on day one and day four before infection. On day five, 100 µL of 2 × 10^6^/mL *A. fumigatus* conidia suspension were injected to the mice via lateral tail vein to cause IA. Two days after infection, mice urine and blood (heparinized) were collected before the mice were euthanized. Potentially infected organs such as liver, spleen and kidney were harvested for tissue fungal culture, histopathological analysis and immunohistochemical staining.

### 4.11. Statistical Analysis

Statistical analyses were performed by SPSS software (version 21.0, IBM Corp., Armonk, NY, USA) and GraphPad software (version 9, Prism, San Diego, CA, USA). Continuous variables were stated as mean and standard deviation (SD). Comparison of absorbance values in multiple groups used one-way ANOVA with post hoc Tukey’s test. A *p* value of less than 0.05 was considered statistically significant.

## Figures and Tables

**Table 1:** mAbs 1D2 and 4E4 recognize only *Aspergillus fumigatus* and *Aspergillus flavus*.

Pathogens	Immunofluorescence	
	1D2	4E4
*Aspergillus fumigatus* (AF293, hyphae)	++	++
*Aspergillus fumigatus* (AF293, geminated conidia)	+/−	+/−
*Aspergillus fumigatus* (AF293, resting conidia)	−	−
*Aspergillus fumigatus* (*Clinical strain 1*)	++	++
*Aspergillus fumigatus* (*Clinical strain 2*)	++	++
*Aspergillus flavus* (hyphae)	++	++
*Aspergillus flavus* (geminated conidia)	+/−	+/−
*Aspergillus flavus* (resting conidia)	−	−
*Aspergillus terreus*	−	−
*Aspergillus niger*	−	−
*Cunninghamella bertholletiae*	−	−
*Rhizopus microspores*	−	−
*Candida albicans*	−	−
*Candida dubliniensis*	−	−
*Candida guilliermondii*	−	−
*Candida glabrata*	−	−
*Candida parapsilosis*	−	−
*Candida tropicolis*	−	−
*Candida lusitaniae*	−	−

Note: ++ strongly positive fluorescence on the cell wall; +/− probably positive fluorescence on the cell wall; − negative fluorescence on the cell wall.

**Table 2:** There are overlapping antigen epitopes recognized by 1D2 and 4E4 as determined by ELISA.

mAbs	Inhibition Percentage of the Following Biotin-Labelled mAbs	
	1D2-Biotin	4E4-Biotin
1D2	53.06% ± 2.32%	37.12% ± 3.24%
4E4	40.92% ± 2.24%	44.29% ± 1.82%

## Data Availability

All data generated or analyzed during this study are included in this published article and its additional files.

## Supplementary Materials

The following are available online at https://www.mdpi.com/article/10.3390/ijms23010252/s1.

## Abbreviations

IA	invasive aspergillosis
PCR	polymerase chain reaction
EORTC/MSGERC	European Organization for Research and Treatment of Cancer and the Mycoses Study Group Education and Research Consortium
GM	galactomannan
mAb	monoclonal antibody
CWFs	cell wall fragments
CWPs	cell wall-related proteins
CBB	Coomassie Brilliant Blue
PBS	phosphate-buffered saline
SDA	PBST: PBS containing 0.1% Tween 20; Sabouraud dextrose agar
SDS/PAGE	sodium dodecyl sulphate polyacrylamide gel electrophoresis
ELISA	enzyme-linked immunosorbent assays
TMB	tetramethybenzidine
TBS	tris buffered saline
