# Supplementary material for: Two Monoclonal Antibodies That Specifically Recognize Aspergillus Cell Wall Antigens and Can Detect Circulating Antigens in Infected Mice
Source: Int J Mol Sci. 2021 Dec 27;23(1):252. doi: 10.3390/ijms23010252 (PMC8745570; doi:10.3390/ijms23010252)
Supplement: Supplementary file 1 [file ijms-23-00252-s001.zip › ijms-1514115-supplementary.pdf]

Antibody	DIC	IF
1D2	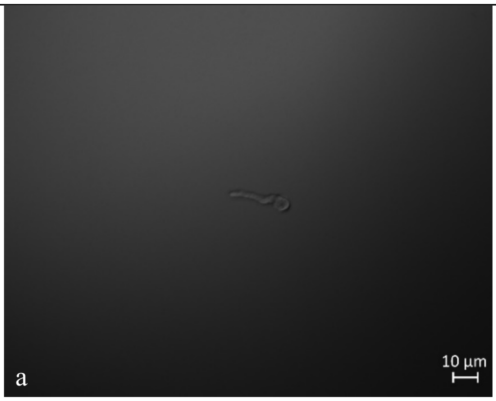	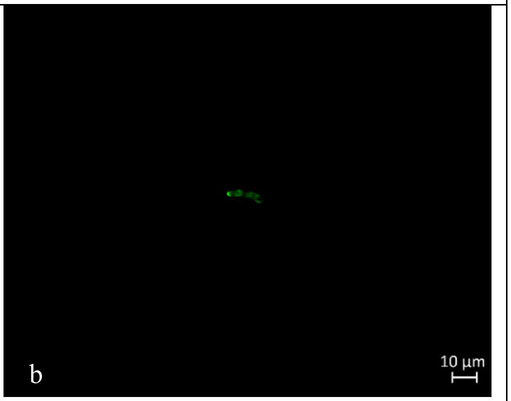
	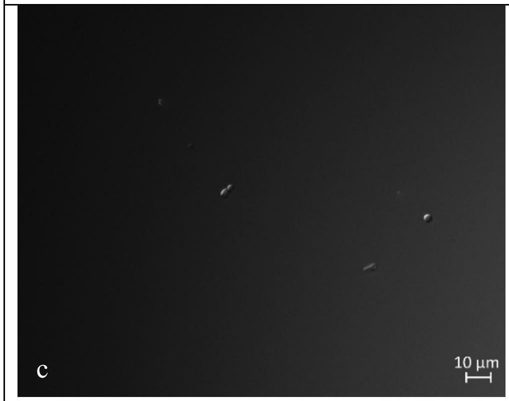	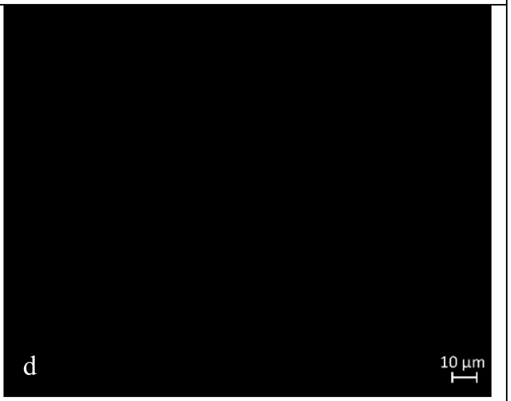
4E4	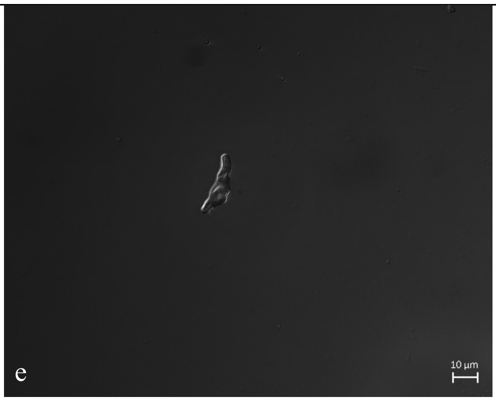	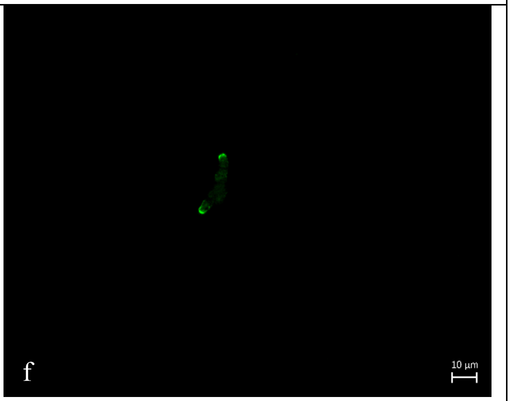
	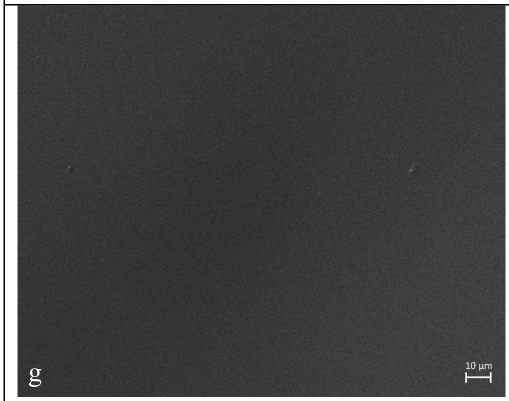	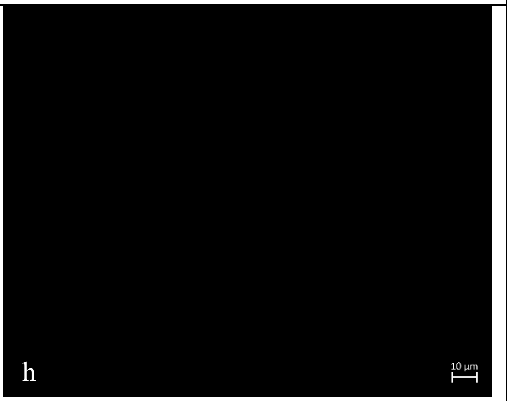

Figure S1 Monoclonal antibodies 1D2 and 4E4 stain geminated conidia and resting conidia of *Aspergillus fumigatus* using immunofluorescence

Both1D2 (a-d) and 4E4 (e-h) showed positive fluorescence on the cell wall of geminated conidia (a, b, e, f) but lacked staining on the resting conidia (c, d, g, h)

DIC: differential interference contrast IF: immunofluorescence

Pathogens	Antibody	DIC	IF
Aspergillus terreus	**1D2**	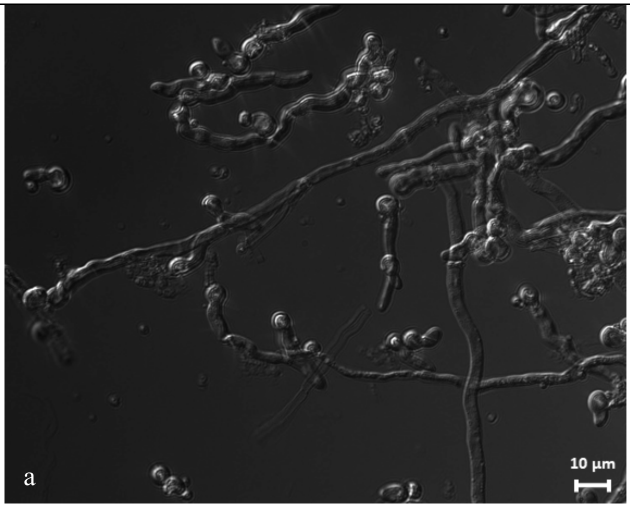	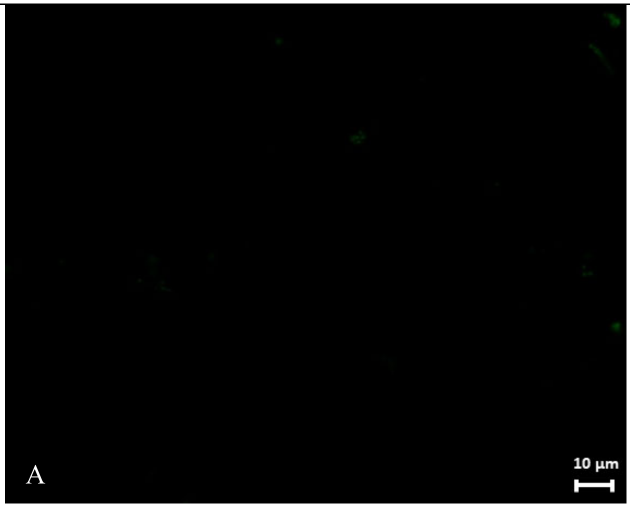
	**4E4**	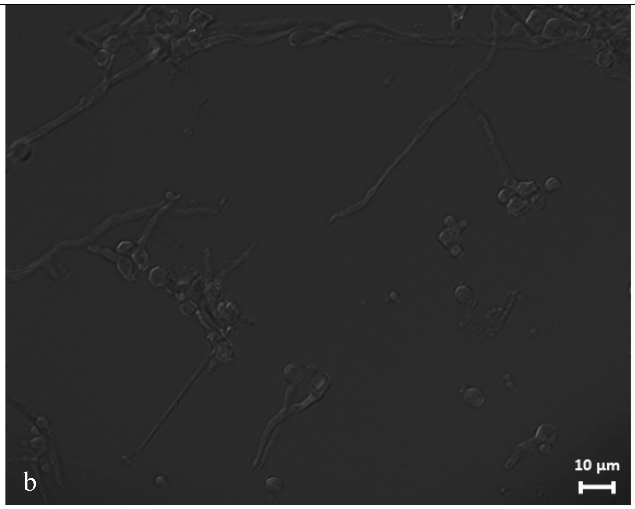	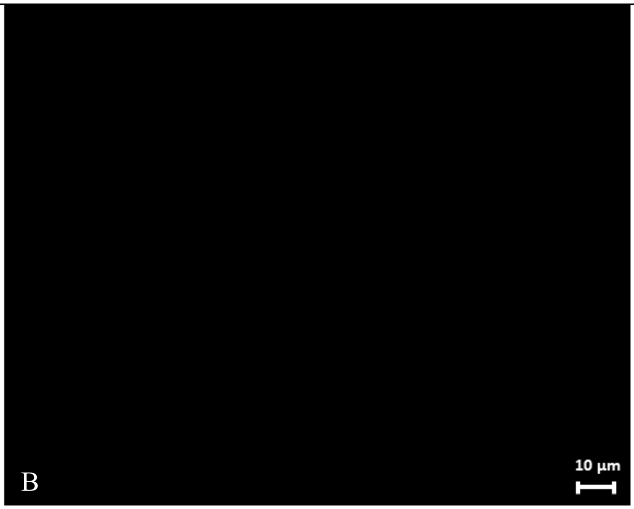

<table>
<tr><th>Pathogens</th><th>Antibody</th><th>DIC</th><th>IF</th></tr>
<tr><td rowspan="2">Aspergillus niger</td><td>1D2</td><td>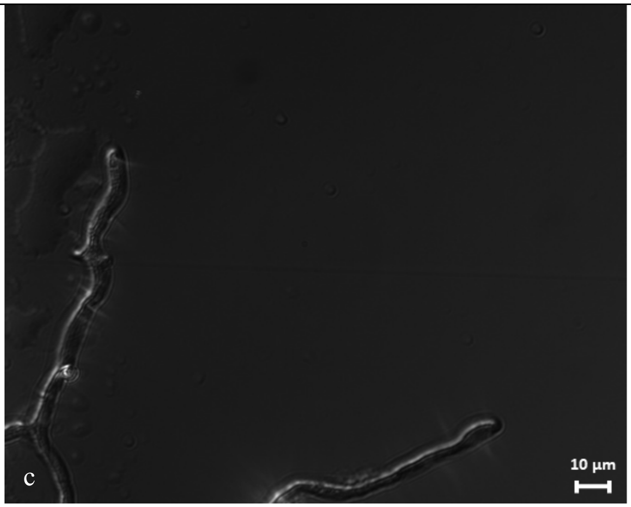</td><td>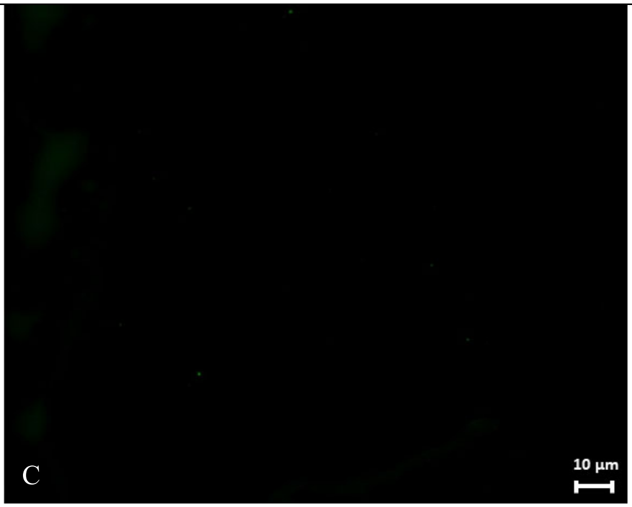</td></tr>
<tr><td>4E4</td><td>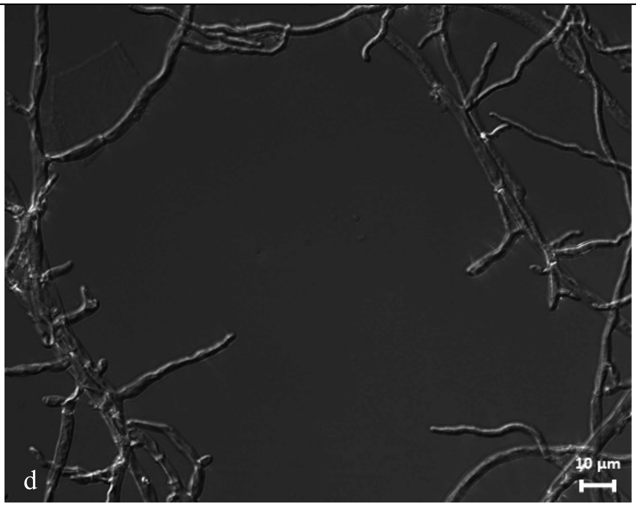</td><td>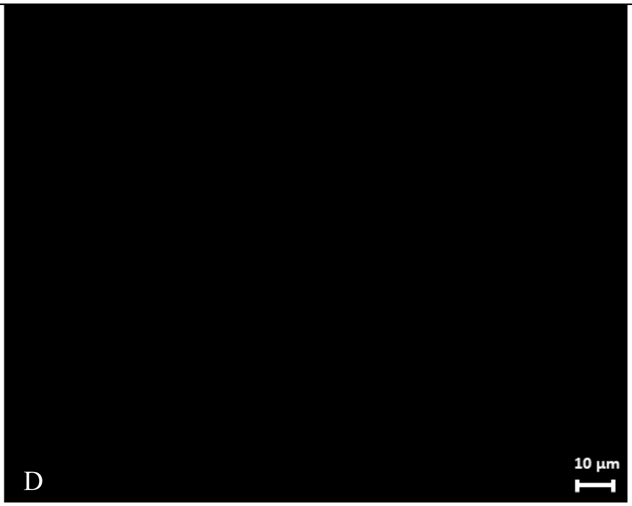</td></tr>
</table>

Pathogens	Antibody	DIC	IF
Cunninghamella bertholletiae	**1D2**	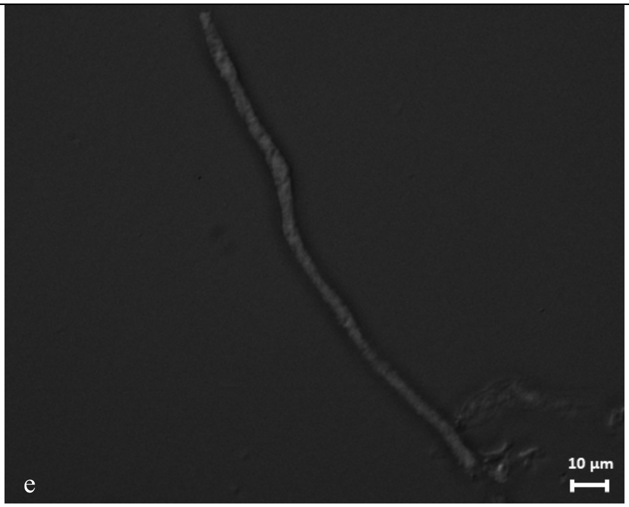	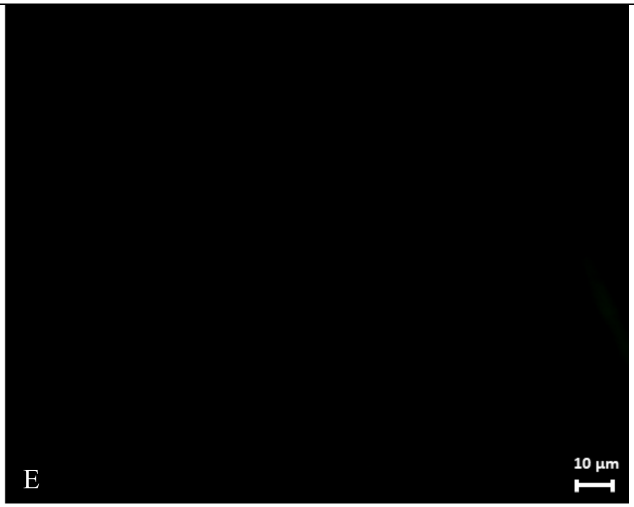
	**4E4**	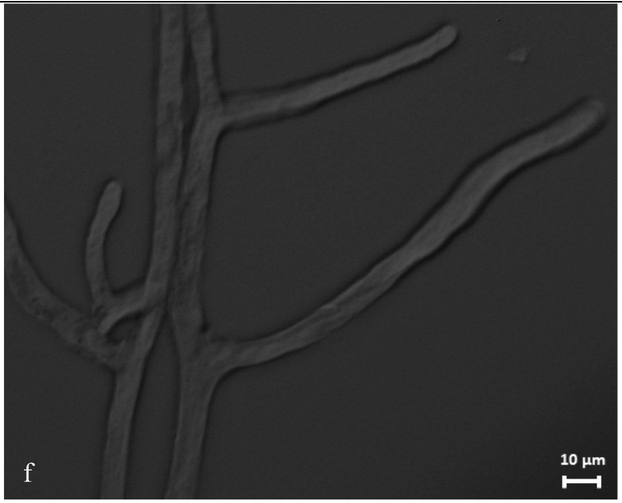	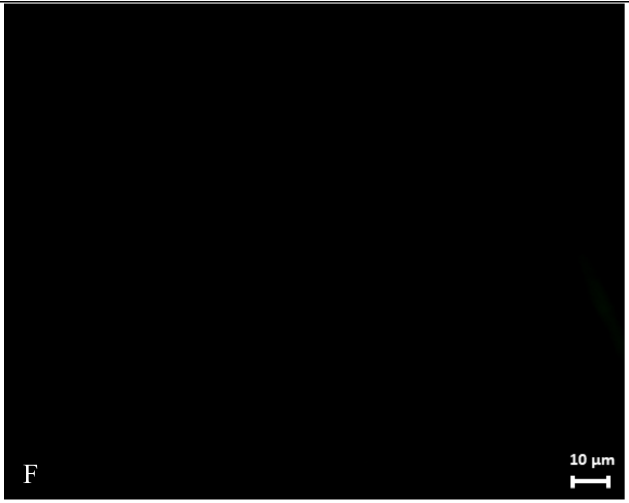

Pathogens	Antibody	DIC	IF
Rhizopus microsporus	**1D2**	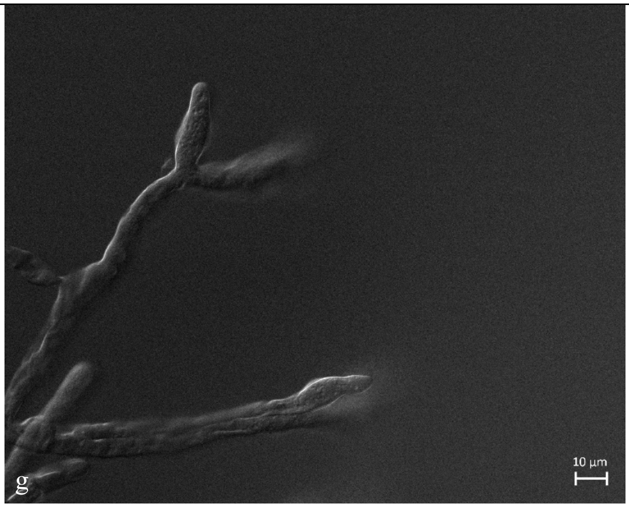	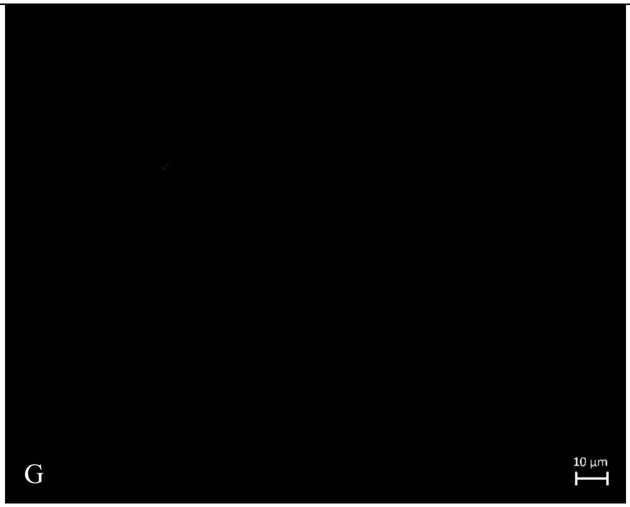
	**4E4**	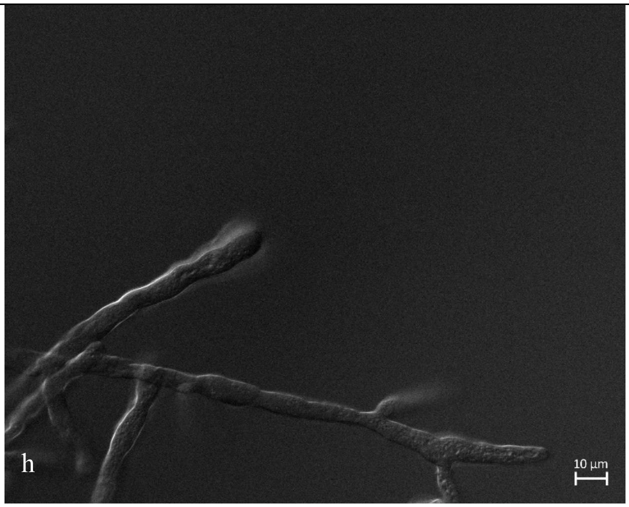	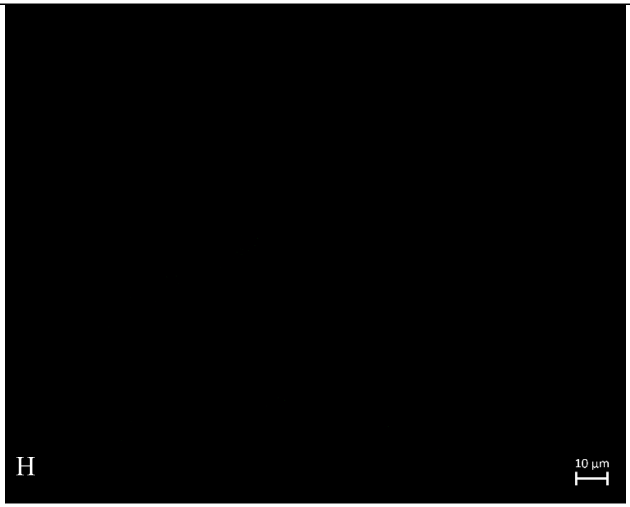

**Pathogens**	**Antibody**	**DIC**	**IF**
Candida albicans	**1D2**	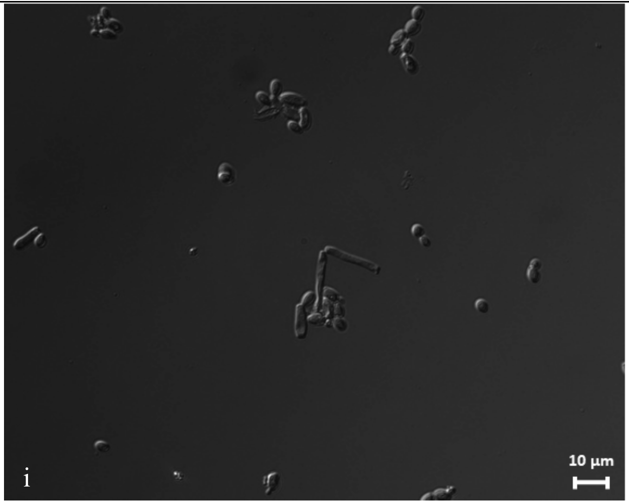	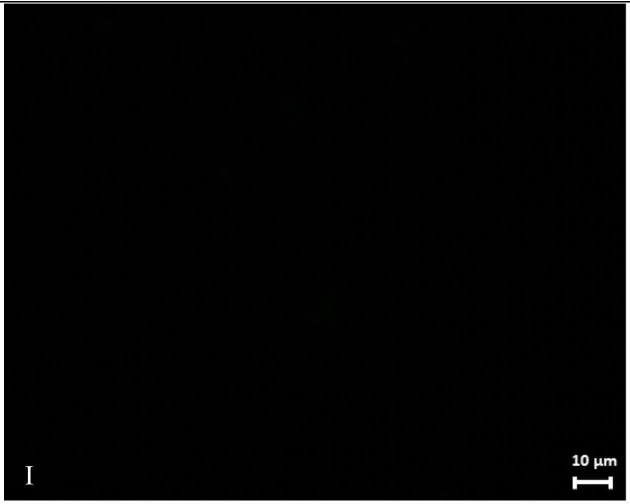
	**4E4**	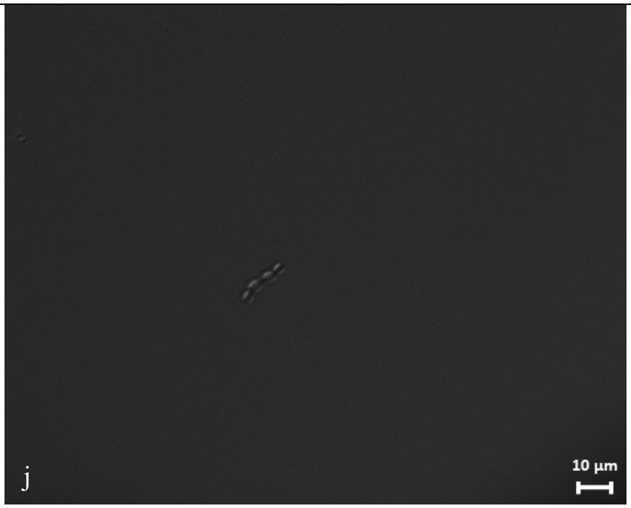	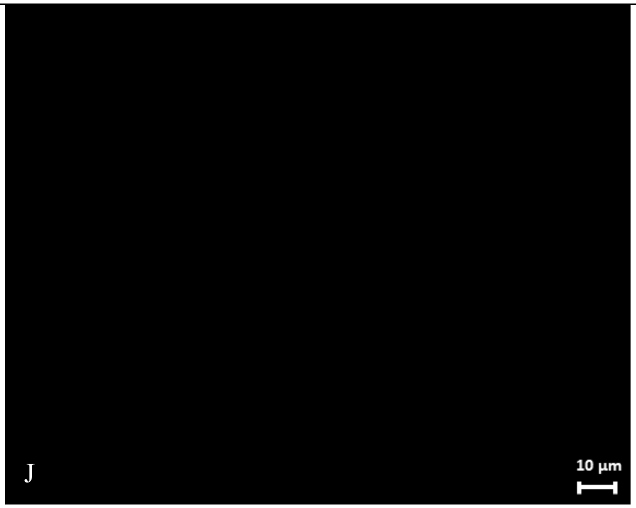

Pathogens	Antibody	DIC	IF
Candida dubliniensis	**1D2**	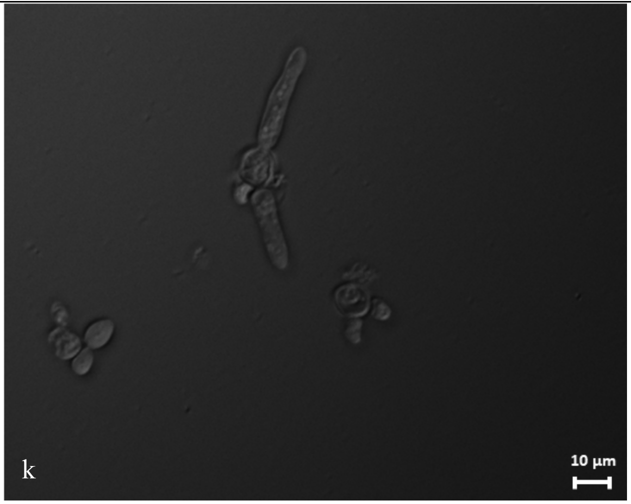	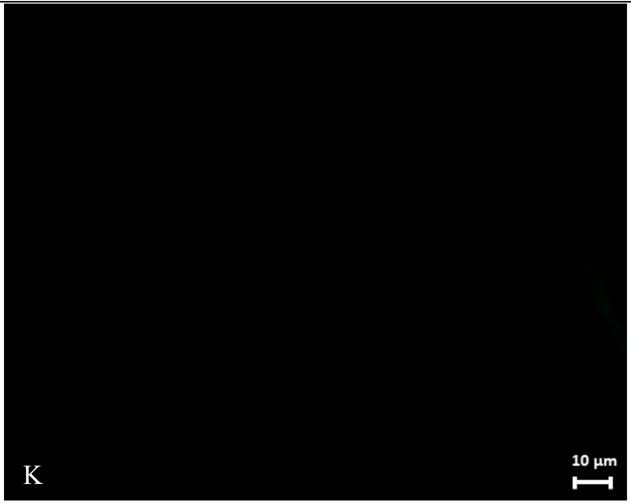
	**4E4**	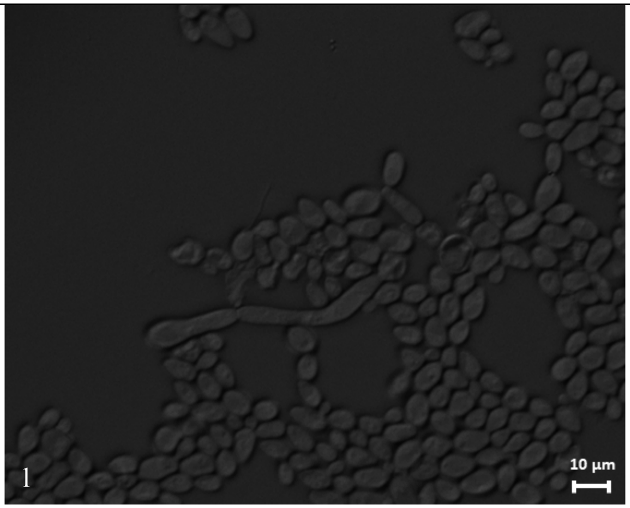	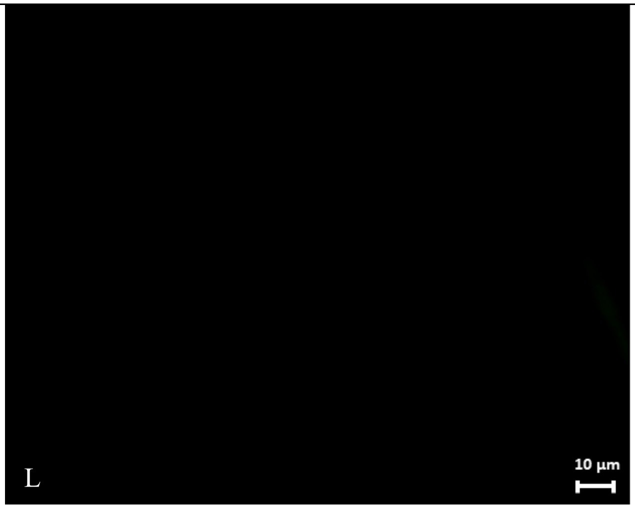

Pathogens	Antibody	DIC	IF
Candida guilliermondii	**1D2**	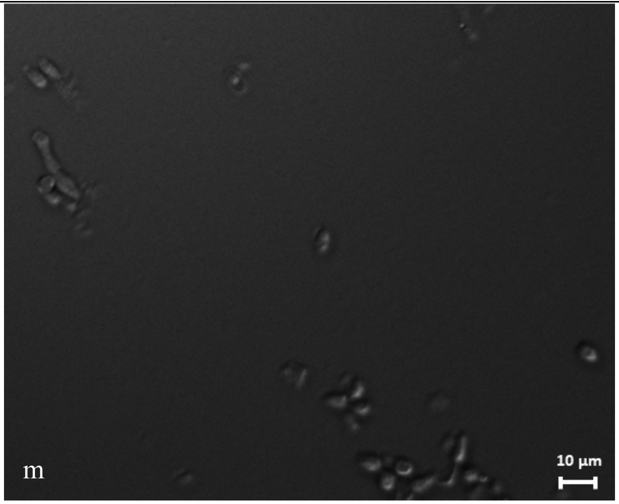	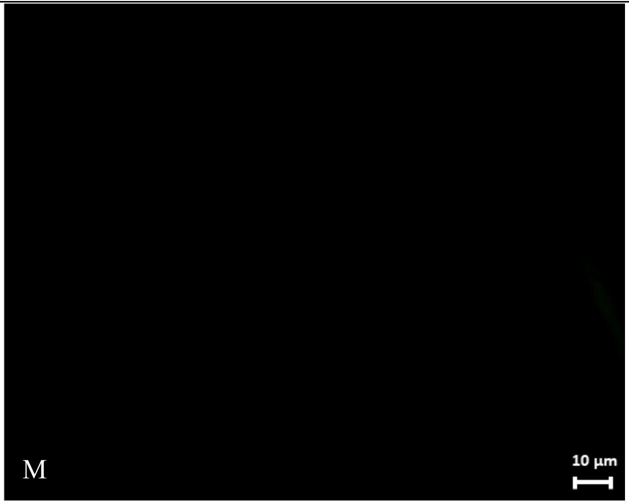
	**4E4**	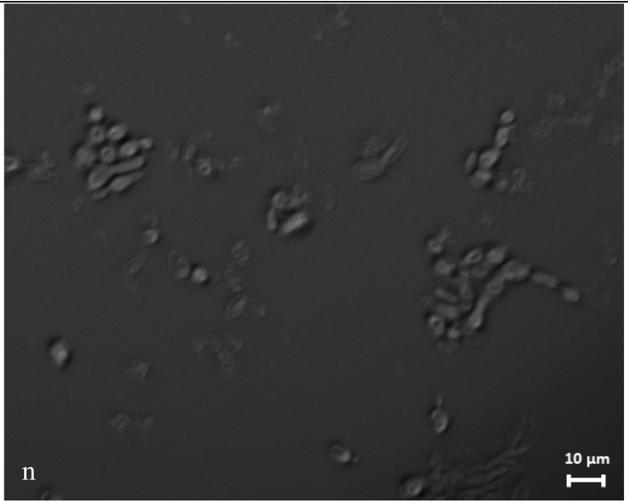	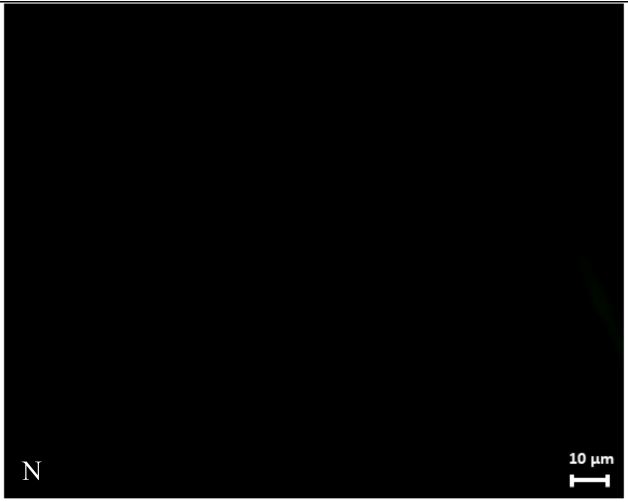

Pathogens	Antibody	DIC	IF
Candida glabrata	**1D2**	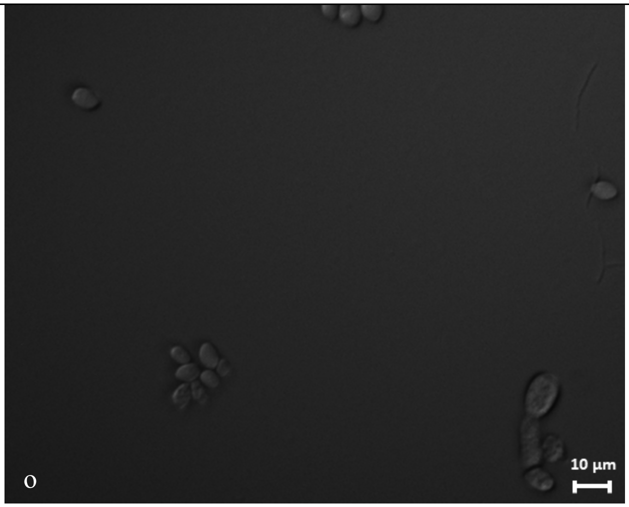	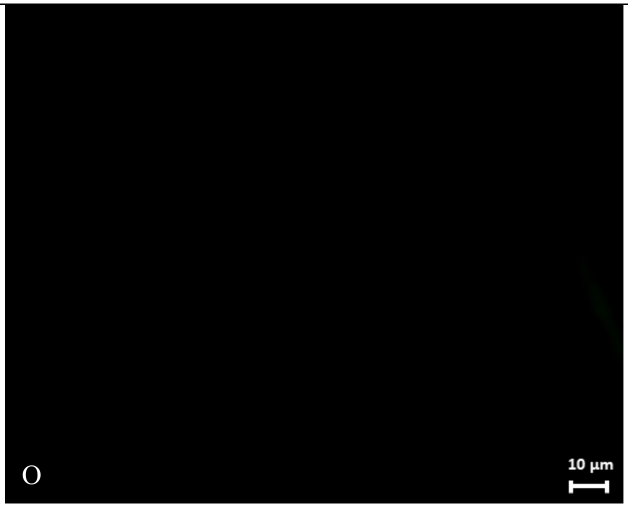
	**4E4**	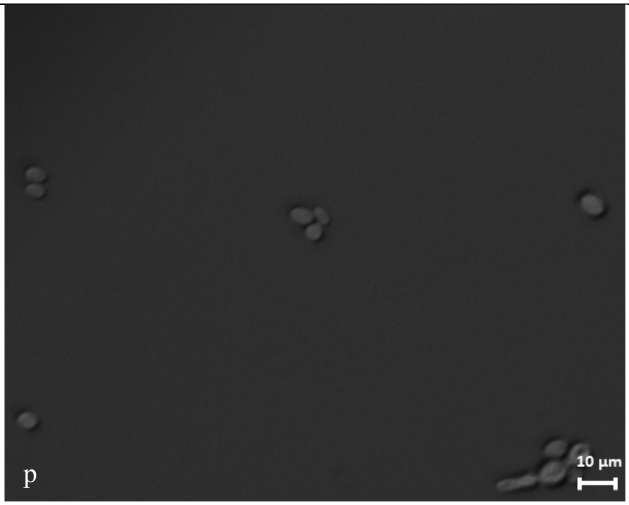	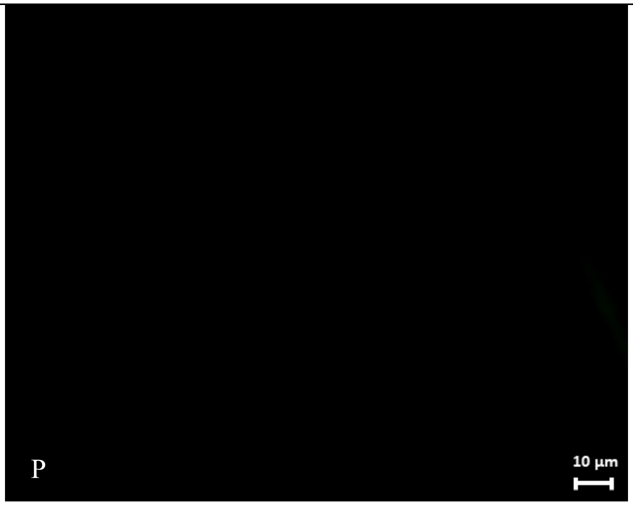

Pathogens	Antibody	DIC	IF
Candida parapsilosis	**1D2**	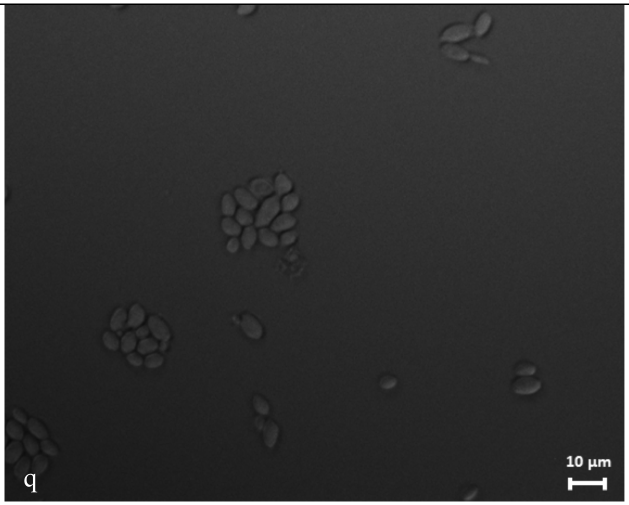	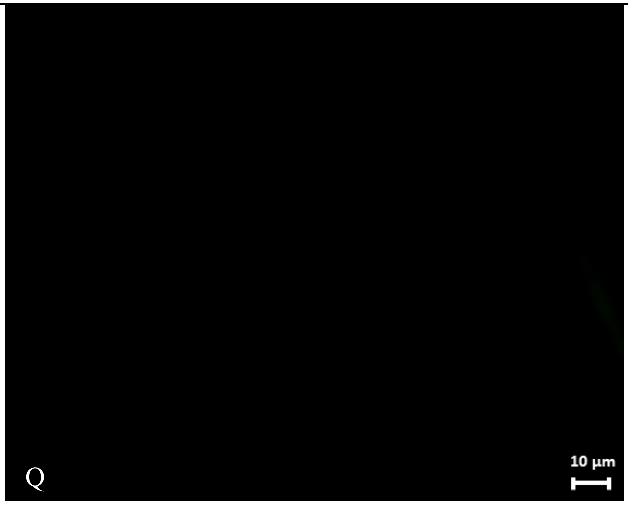
	**4E4**	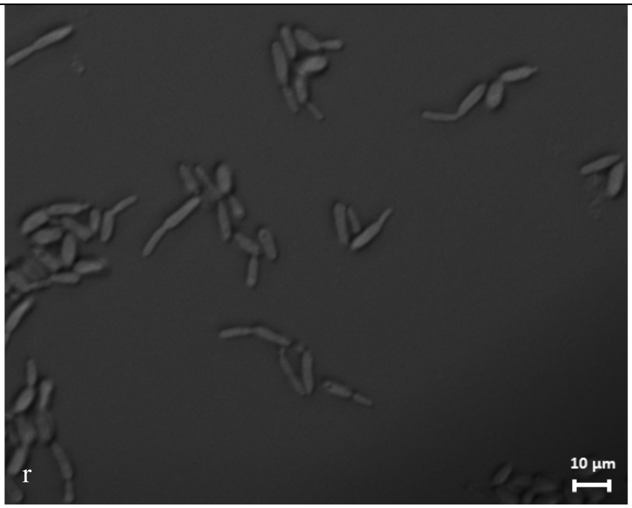	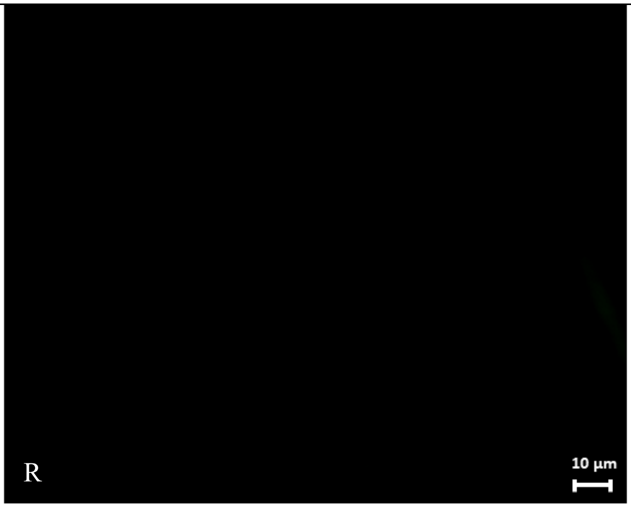

Pathogens	Antibody	DIC	IF
Candida tropicolis	**1D2**	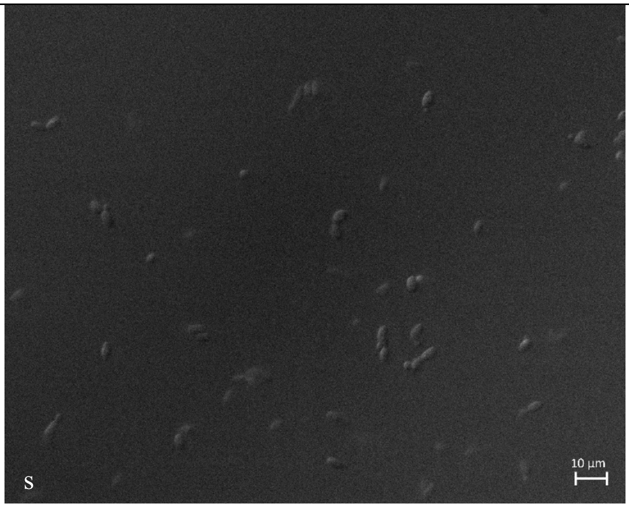	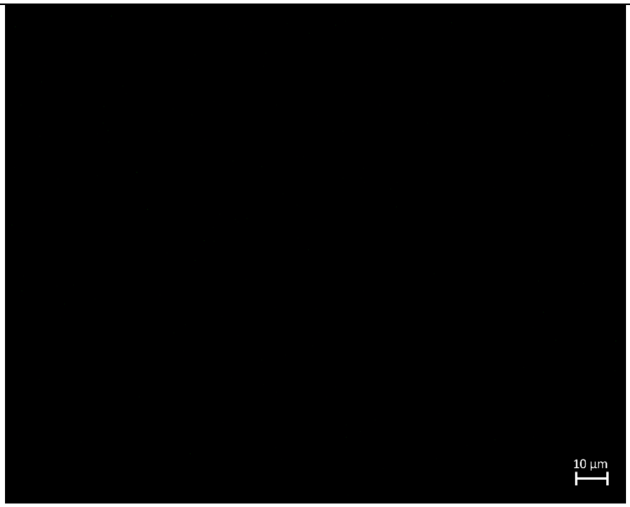
	**4E4**	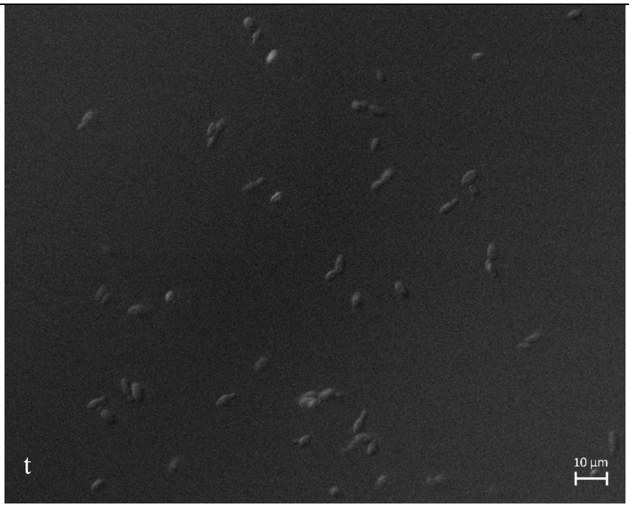	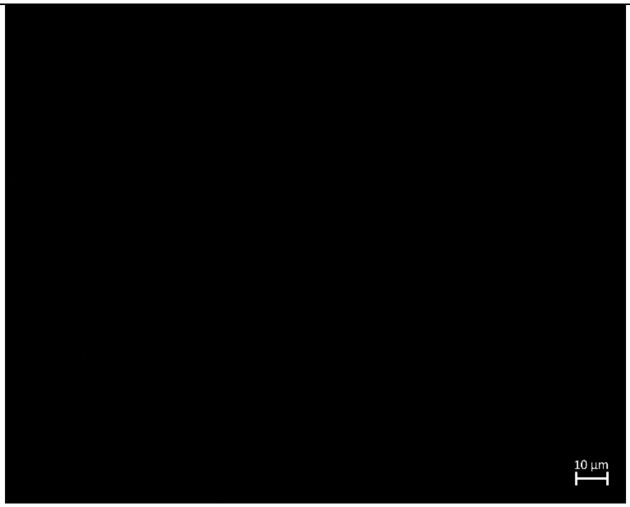

Pathogens	Antibody	DIC	IF
Candida lusitaniae	**1D2**	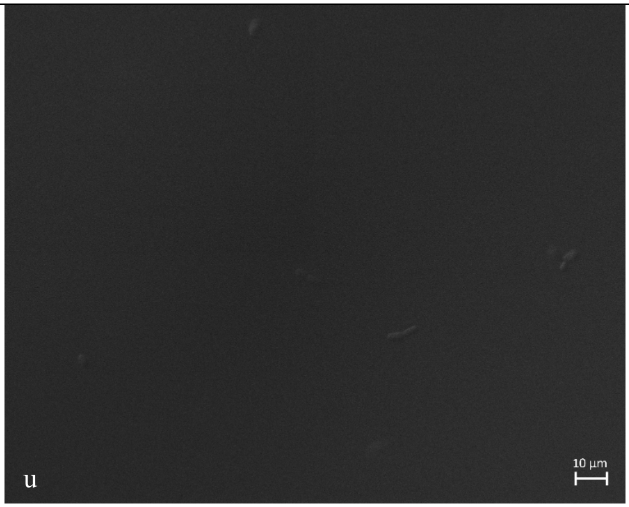	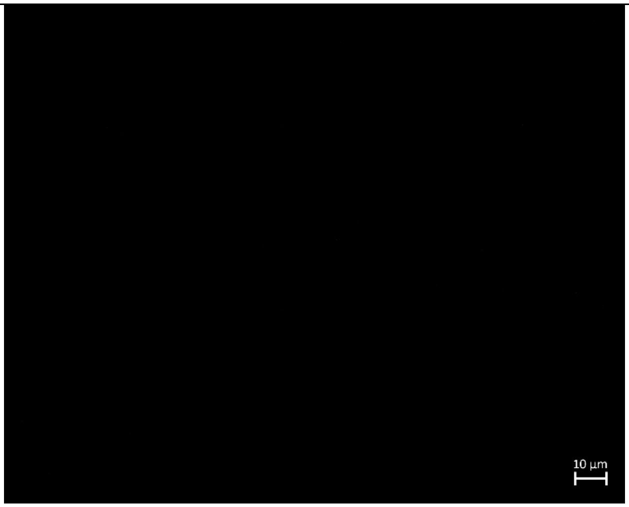
	**4E4**	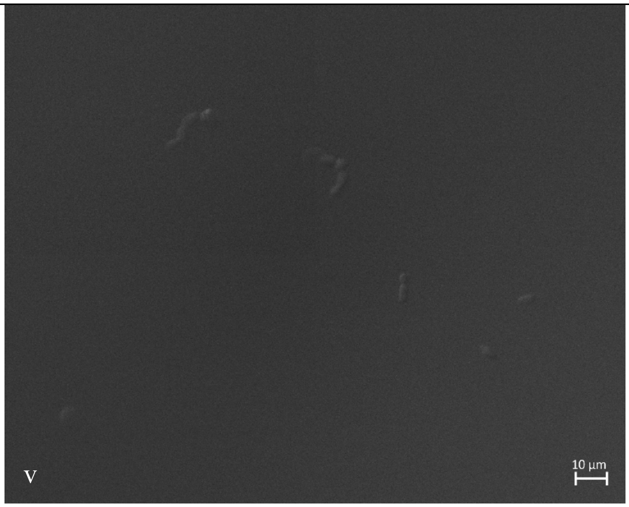	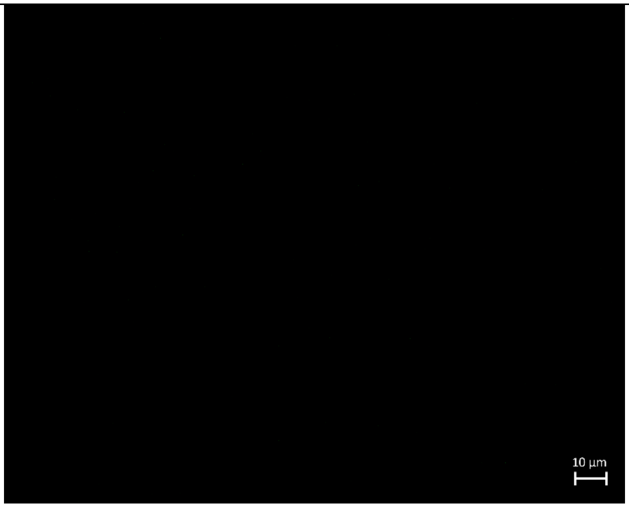

Figure S2 Monoclonal antibodies 1D2 and 4E4 stain other fungi using immunofluorescence

Both 1D2 and 4E4 showed negative fluorescence on the cell wall of *Aspergillus terreus* (a-b, A-B), *Aspergillus niger* (c-d, C-D), *Cunninghamella bertholletiae* (e-f, E-F), *Rhizopus microspores* (g-h, G-H), *Candida albicans* (i-j, I-J), *Candida dubliniensis* (k-l, K-L), *Candida guilliermondii* (m-n, M-N), *Candida glabrata* (o-p, O-P), *Candida parapsilosis* (q-r, Q-R), *Candida tropicolis* (s-t, S-T), *Candida lusitaniae* (u-v, U-V),

DIC: differential interference contrast IF: immunofluorescence

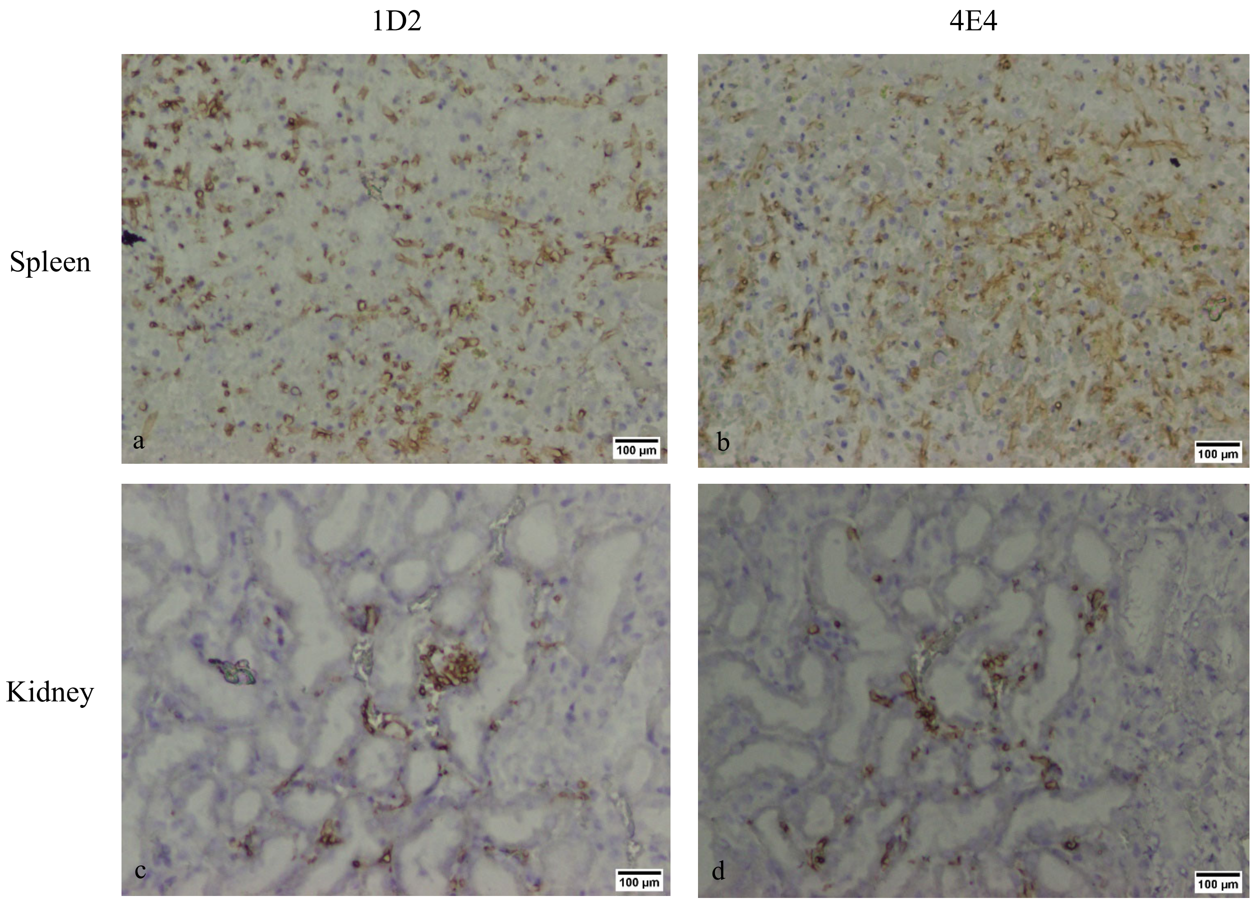

Figure S3 ***Aspergillus*** **monoclonal antibodies 1D2 and 4E4 show immunohistochemical staining in formalin-fixed paraffin spleen and kidney sections from** ***A. fumigatus*** **infected mice.** Immunohistochemistry staining indicated both monoclonal antibody 1D2 (a, c) and 4E4 (image b, d) showed significant staining on the hyphal wall in spleen (a, b) and kidney (image c, d) infection tissues.

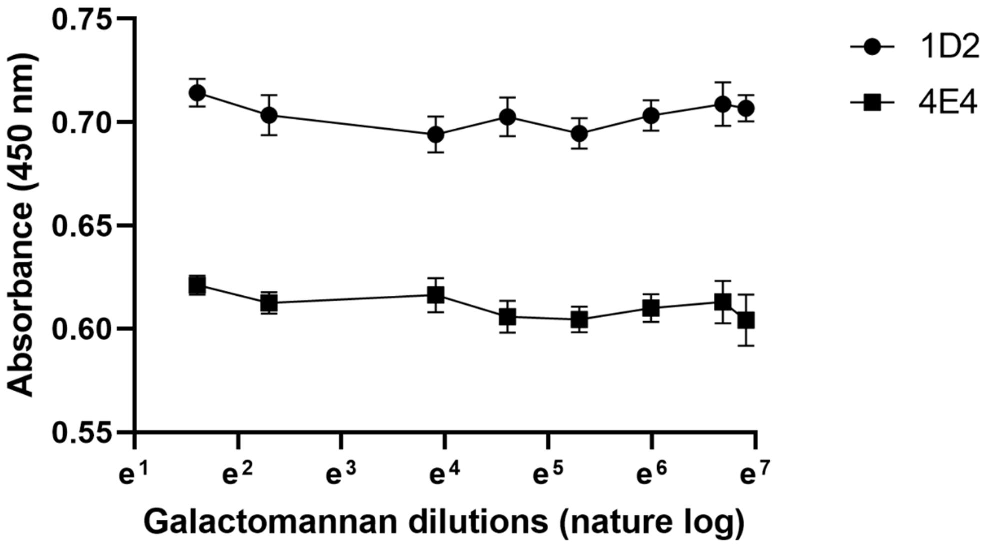

Figure S4 Competitive ELISA between the galactomannan and immobilized *A. fumigatus* antigens. The binding of either 1D2 or 4E4 to the immobilized *A. fumigatus* CWFs did not inhibited by the addition of various concentrations of galactomannan, which indicated that both 1D2 and 4E4 do not identify the carbohydrate epitopes on the galactomannan

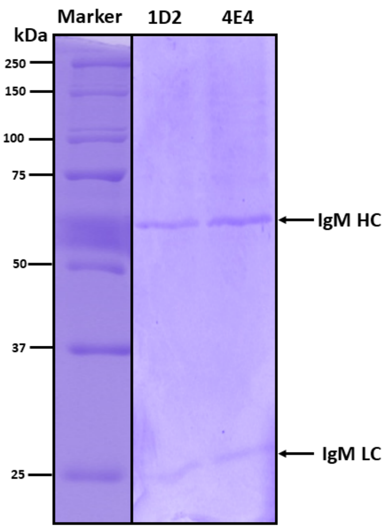

Figure S5 SDS-PAGE analysis (reducing conditions) of monoclonal IgM 1D2 and 4E4. Culture supernatants of hybridoma 1D2 clone and 4E4 clone were purified using a LigaTrap mouse IgM purification resin column. The purified 1D2 and 4E4 were analysed by SDS-PAGE with Coomassie Brilliant Blue staining. IgM HC, heavy chain of IgM; IgM LC, light chain of IgM.
